# Development of the China’s list of ambulatory care sensitive conditions (ACSCs): a study protocol

**DOI:** 10.1186/s41256-024-00350-5

**Published:** 2024-03-19

**Authors:** Jianjian Wang, Dong Roman Xu, Yan Zhang, Hongqiao Fu, Sijiu Wang, Ke Ju, Chu Chen, Lian Yang, Weiyan Jian, Lei Chen, Xiaoyang Liao, Yue Xiao, Ruixian Wu, Mihajlo Jakovljevic, Yaolong Chen, Jay Pan

**Affiliations:** 1 HEOA Group, West China School of Public Health and West China Fourth Hospital, Sichuan University, Chengdu, China; 2 School of Health Management, Southern Medical University, Guangzhou, China; 3 School of Medicine and Health Management, Tongji Medical College, Huazhong University of Science and Technology, Wuhan, China; 4 Department of Health Policy and Management, School of Public Health, Peking University Health Science Center, Beijing, China; 5 Vanke School of Public Health, Tsinghua University, Beijing, China; 6 School of Public Health and Preventive Medicine, Monash University, Melbourne, Australia; 7 School of Health Management, Fujian Medical University, Fujian, China; 8 School of Public Health, Chengdu University of Traditional Chinese Medicine, Chengdu, China; 9 Department of Neurology, West China Hospital, Sichuan University, Chengdu, China; 10 General Practice Ward/International Medical Center Ward, General Practice Medical Center, West China Hospital, Sichuan University, Chengdu, China; 11 China National Health Development Research Center, Beijing, China; 12 Center for Health Statistics and Information, National Health Commission, Beijing, China; 13 Institute of Advanced Manufacturing Technologies, Peter the Great St. Petersburg Polytechnic University, St. Petersburg, Russia; 14 Institute of Comparative Economic Studies, Faculty of Economics, Hosei University, Tokyo, Japan; 15 Department of Global Health Economics and Policy, Faculty of Medical Sciences, University of Kragujevac, Kragujevac, Serbia; 16 Research Unit of Evidence-Based Evaluation and Guidelines, Chinese Academy of Medical Sciences (2021RU017), School of Basic Medical Sciences, Lanzhou University, Lanzhou, China; 17 World Health Organization Collaborating Center for Guideline Implementation and Knowledge Translation, Lanzhou University, Lanzhou, China; 18 Institute for Healthy Cities and West China Research Center for Rural Health Development, Sichuan University, Chengdu, China

**Keywords:** Ambulatory care sensitive conditions, Avoidable hospitalizations, Primary care, Quality of health care

## Abstract

**Background:**

The hospitalization rate of ambulatory care sensitive conditions (ACSCs) has been recognized as an essential indicator reflective of the overall performance of healthcare system. At present, ACSCs has been widely used in practice and research to evaluate health service quality and efficiency worldwide. The definition of ACSCs varies across countries due to different challenges posed on healthcare systems. However, China does not have its own list of ACSCs. The study aims to develop a list to meet health system monitoring, reporting and evaluation needs in China.

**Methods:**

To develop the list, we will combine the best methodological evidence available with real-world evidence, adopt a systematic and rigorous process and absorb multidisciplinary expertise. Specific steps include: (1) establishment of working groups; (2) generations of the initial list (review of already published lists, semi-structured interviews, calculations of hospitalization rate); (3) optimization of the list (evidence evaluation, Delphi consensus survey); and (4) approval of a final version of China’s ACSCs list. Within each step of the process, we will calculate frequencies and proportions, use descriptive analysis to summarize and draw conclusions, discuss the results, draft a report, and refine the list.

**Discussion:**

Once completed, China’s list of ACSCs can be used to comprehensively evaluate the current situation and performance of health services, identify flaws and deficiencies embedded in the healthcare system to provide evidence-based implications to inform decision-makings towards the optimization of China’s healthcare system. The experiences might be broadly applicable and serve the purpose of being a prime example for nations with similar conditions.

## Introduction

The quality of healthcare services has been recognized as the core element of healthcare system construction under the concept of value-based healthcare in the new era [1]. As such, constant evaluation of medical quality has become essential to facilitate the enhancement of medical service delivery as well as to improve the utilization efficiency of medical resources under the universal goal of human health promotion. However, measuring health systems performance has remained ambiguous. In order to evaluate the quality of healthcare services, David D. Rutstein et al. in 1976 first proposed the adoption of potentially avoidable diseases as negative indicators to reflect accessibility and quality of healthcare [2]. These diseases, known as ambulatory care sensitive conditions (ACSCs), could have been avoided via the provision of timely and effective ambulatory services. Early detection and treatment play a pivotal role in disease management promotion in terms of controlling acute onset episodes of these conditions, thus further reducing the risk of potentially avoidable hospitalizations for patients diagnosed with these diseases [3–5].

Different countries and health systems define ACSCs differently. In 1993, the Institute of Medicine (IOM) listed 19 diseases, including hypoglycemia, dehydration, hypertension, and diabetes, as ACSCs [6]. Subsequently, the Agency for Healthcare Research and Quality (AHRQ) [7], the National Institute for Health and Care Excellence (NICE) [8], the Canadian Institute for Health Information (CIHI) [9], the Organization for Economic Co-operation and Development (OECD) [10] and other research institutes for medical quality surveillance all published their own lists of ACSCs. Despite different versions of ACSCs lists, a universal consensus has been reached that the list of ACSCs should contain three major types of diseases, namely chronic diseases such as diabetes and hypertension, for which high-quality health management is key to mitigating acute exacerbation and subsequent complications, thus further avoiding hospitalizations; acute diseases like dehydration symptoms and gastroenteritis, which can be appropriately controlled through timely and effective out-of-hospital treatment as an early-stage intervention to alleviate symptoms; and infectious diseases like measles and mumps, which can be reduced by vaccination to avoid disease-induced hospitalization.

Increased attention has been raised up among worldwide nations to address issues in relation to the onsets of ACSCs [11–14], while major developed countries have adopted ACSCs as an essential indicator to reflect the quality and accessibility of medical service delivery for health surveillance purposes at the governmental level [15–19]. However, the occurrence of ACSCs is influenced by a combination of individual factors, health system factors, and socioeconomic factors [20, 21]. Studies have reported large outcome disparities when different versions of ACSCs lists are used to assess potentially avoidable hospitalizations [8, 22]. To date, all relevant studies conducted by Chinese scholars used lists produced by other nations due to the lack of a well-established ACSCs list tailored for the context of China’s healthcare system [23, 24]. Directly utilizing ACSCs lists from other nations may not be able to provide a scientifically precise assessment of health services in China. In particular, the primary healthcare system in China is yet to be fully developed at this stage, with inpatient services in hospitals exhibiting significant differences compared to those in countries such as the United Kingdom and the United States. Taking angina patients as an example, those in the US may be able to avoid hospitalization through treatment from family doctors or general practitioners in the UK, whereas those in China are more likely to be hospitalized in tertiary hospitals [25].

The constantly aging population, expanded disease spectrum, along with increased health insurance coverage has posed unprecedented challenges to Chinese residents as well as the whole society, as reflected by the total number of hospital admissions across China which sharply increased from 95.24 million in 2010 to 230.13 million in 2020, in addition to the per capita medical costs of inpatients which increased from 6525.6 yuan in 2010 to 10,619.2 yuan in 2020 [26]. Under such challenging circumstances, minimizing avoidable hospitalizations through early outpatient services has become crucial for optimizing medical resource utilization under constrained conditions. As such, developing China’s unique version of ACSCs list should be highlighted as an urgent task to meet the demands of Chinese residents under the context of China’s healthcare system. Inspired by the methodology used to develop evidence-based clinical practice guidelines, and referenced some of the key steps [27–29], this study aims to develop a list of ACSCs focusing on health care conditions uniquely embedded among China’s population groups, in order to provide evidence-based implications to inform decision-makings towards the optimization of China’s healthcare system.

## Methods

**Fig. 1:**
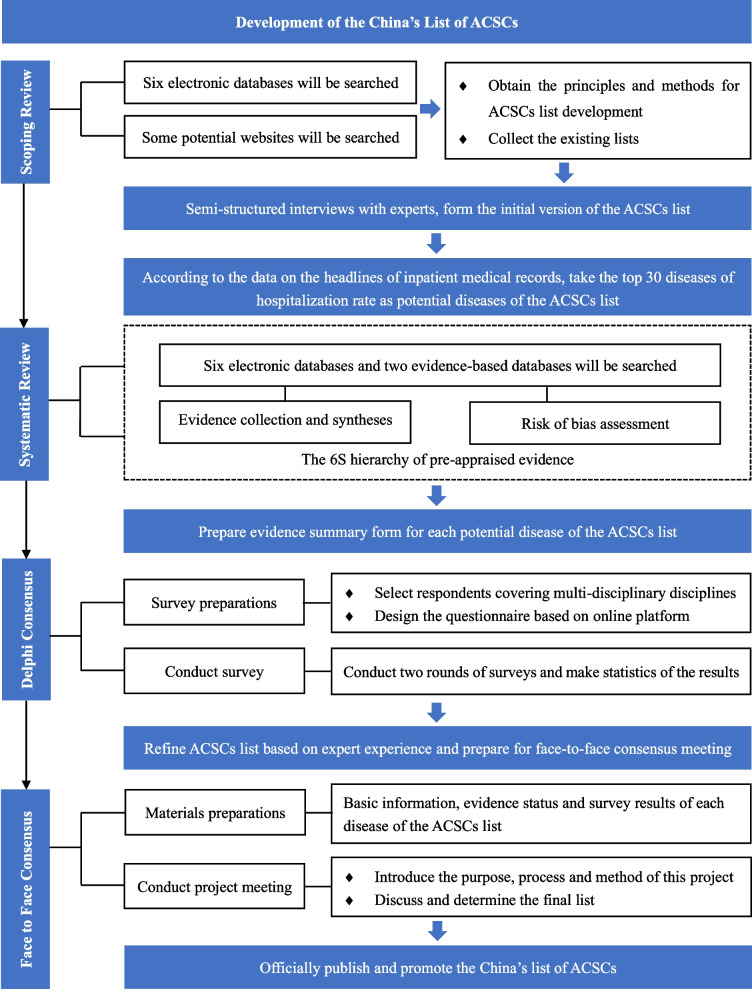
Flowchart of the development process

To develop China’s context-specific list of ACSCs, we will follow a proven multistep process used in the development of similar lists [5–10, 30–35], including scoping review, semi-structured interviews, descriptive study, systematic review, Delphi consensus survey and face-to-face consensus. Table 1 describes the multistep development process of the China’s ACSCs list, which includes: (1) establishment of working groups; (2) generations of the initial list; (3) optimization of the list; and (4) approval of the final list version. Figure 1 illustrates the development process.

**Table 1:** Description of the multistep development process

Steps and sub-steps	Establishment of working groups	Generation of the initial list			Optimization of the list		Approval of the final list version
		Review of published lists	Semi-structured interviews	Calculations of hospitalization rate	Evidence evaluation	Delphi consensus survey	
Main objective	To identify individuals who are relevant to participant in the project	To develop the initial version of the ACSCs list			To collect and synthesize the evidence of each disease in the ACSCs list	To define the list of diseases to be included in the list	To approve the final version of the list
Study design	–	Scoping review	Semi-structured interviews	Descriptive study	Systematic review	Delphi consensus survey	Face-to-face consensus
Participants	All participants	Evidence Review Team (6 participants)	Advisory Group (7 participants)	Evidence Review Team (2 participants)	Evidence Review Team (8 participants)	Delphi Panel (21–29 participants)	Advisory Group (7 participants)
			Coordination Team (2 participants)		Coordination Team (2 participants)	Coordination Team (2 participants)	Delphi Panel (21–29 participants)
							Coordination Team (2 participants)
Main outcome	Advisory Group (7 participants)	Potential diseases in the published lists	Participants’ views and experiences on ACSCs	The top 30 diseases of hospitalization rates in China	The evidence status of each potential disease in the list	Diseases considered appropriate in China’s list of ACSCs	The final version of China’s list of ACSCs
	Delphi Panel (21–29 participants)						
	Evidence Review Team (8 participants)						
	Coordination Team (2 participants)

### Step I: establishment of working group

The primary aim of this step is to identify individuals who are relevant to participant in the project. The working group will include: (1) an Advisory Group with seven participants; (2) a Delphi Panel with 21–29 participants; (3) an Evidence Review Team with eight participants; and (4) a Coordination Team with two participants. When determining the number of members in each group, we have taken into account both practical feasibility and the distribution of personnel in previous studies' working groups and clinical practice guidelines.

#### Advisory group

The Advisory Group is a multidisciplinary group including seven researchers who have substantial experience in health policy making or evidence-based medicine, and have certain degrees of understanding of ACSCs. Specifically, they will be responsible for (1) establishing other working groups; (2) managing conflicts of interest; (3) approving development proposals; (4) examining and approving the final report; and (5) supervising the development process and providing advice and guidance when necessary.

#### Delphi panel

The Delphi Panel will incorporate 21–29 multidisciplinary representatives from across the country, with specialties in diverse areas such as clinical medicine, epidemiology, evidence-based medicine, health policy and health economics. At least 80% of these members are clinical workers, with half being general or primary care physicians. Specifically, they will be responsible for (1) voting until consensus is reached on the list's contents; and (2) finalizing the report.

#### Evidence review team

The Evidence Review Team will incorporate eight researchers with experiences in evidence-based medicine. Their primary responsibilities include: (1) searching, evaluating, synthesizing, and grading evidence; and (2) creating summary tables to describe evidences identified.

#### Coordination team

The Coordination Team is in charge of managing the project at different stages of implementation. They will coordinate the list development process and ensure its completion according to the established timeline. Specifically, the Coordination Team will be responsible for (1) coordinating the work of other working groups; (2) drafting and developing the protocol; (3) conducting semi-interviews, surveys and organizing consensus meetings; (4) documenting the entire list development process in details; and (5) preparing the preliminary draft of the report.

### Step II: Generations of the initial list

The main purpose of this step is to develop the initial list, which is accomplished by reviewing published lists, further supplementing them through expert interviews, and selecting the diseases with the highest hospitalization rates in China as potential diseases of the China’s ACSCs list. It includes three sub-steps: (1) review of published lists by scoping review; (2) semi-structured interviews to understand participants’ views and experiences on ACSCs; and (3) calculation of hospitalization rates by a descriptive study to identify the top 30 diseases with highest hospitalization rates in China.

#### Review of published lists

We will search, review and classify the published literature and reports related to ACSCs and their development, collect the existing versions of lists, and obtain the principles and methods of ACSCs list development, specifically the criteria for including or excluding diseases of the list. These contents will also serve as key conditions and content in the subsequent semi-structured interviews, Delphi surveys, and face-to-face consensus.

The Evidence Review Team will systematically search the existing literature and reports regarding ACSCs based on six commonly used Chinese and English databases, including China National Knowledge Internet (CNKI), China Biology Medicine disc (CBMdisc), Wanfang Database, MEDLINE (via PubMed), Scopus, and Web of Science. There will be no publication status restrictions. To sort out the development methods of the ACSCs list and extract relevant disease information, e.g., specific types and codes, we will also conduct additional search on Google and the official websites of major international medical quality research institutions, including AHRQ, NICE, CIHI, OECD and World Health Organization (WHO). To minimize potential bias, literature searching, screening, and information extraction will be independently performed by two reviewers in pairs, and any disagreements will be resolved through discussion. Detailed inclusion and exclusion criteria are presented in Table 2.

**Table 2:** Inclusion and exclusion criteria

Inclusion	Exclusion
Studies about the development of ACSCs	Full-text unavailable due to specific reasons
Studies about healthcare quality evaluation with ACSCs	Duplicates
Published language: English and Chinese	Study protocols and conference papers
Published year: no restriction	

#### Semi-structured interviews

To gather participants' views and experiences on ACSCs, as well as to explore potential relevant diseases for the initial version of the ACSCs list, semi-structured interviews will be conducted with relevant researchers of ACSCs, particularly focusing on clinical workers and general or primary care physicians. We will identify participants through convenience sampling with the support of the Advisory Group, contact them via emails, and conduct face-to-face or online interviews. The sample size will be guided by “information power” (a model for assessing the adequacy of sample sizes in qualitative research) [36, 37].

The interview outline will be determined by referring to previous studies, and in order to gain a wealth of information, the outline will be sent to the participants in advance. The main points of the interviews will include: (1) whether the diseases identified in the preceding step belong to China’s list of ACSCs, including whether timely and effective outpatient services can prevent potential avoidable hospitalizations, whether they can alleviate the condition and avoid aggravation once an incident occurs, as well as whether 48-h hospitalization is necessary when there is a hospitalization indication; (2) what other diseases can be added to the China’s ACSCs list that meet the above criteria; and (3) any additional matters concerning the development of the ACSCs list. Pre-interviews will be conducted when necessary to guarantee the successful completion of the interview.

Each interview is scheduled for approximately 40 to 50 min and will be recorded with the participant's consent for subsequent transcription. The interview records will be sent to the participants for check and approval. Since the views and experiences of participants are qualitative variables, we will summarize and draw conclusions based on content analysis [38]. The Coordination Team will review and verify the results. If a new disease is proposed by more than half of the participants, it will be added to the initial list. In case of disagreements, they will consult with the Advisory Group.

#### Calculations of hospitalization rate

By analyzing the data from the cover pages of inpatient medical records, which include patients' demographic information, admission details, and discharge information, we will identify the top 30 diseases with the highest hospitalization rates in China. The formula for calculating hospitalization rates is as follows:

$$Hospitalization\,rates=\frac{Number\,of\, hospitalizations\, for\, a\, certain\, disease}{Number\, of\, adjusted\, population}\times 100\%$$

Since the hospitalization rates are quantitative variables, we will calculate absolute frequencies and proportions. Those diseases that are both found in the top 30 hospitalization rates list and the initial version of the list will be regarded as potential diseases. If feasible, the economic burden of the diseases will also be calculated and included as a criterion for selection.

In this step, we conjecture that certain diseases with high hospitalization rates may not necessarily be ACSCs and that certain diseases may have highly variable hospitalization rates due to large differences in treatment, both of which need to be determined in the subsequent steps.

### Step III: Optimization of the list

The published list of ACSCs is primarily based on expert opinions rather than available evidence. Previous research indicates that selection of defined ACSCs should be based on evidence rather than expert view [34]. This step also incorporates the “evidence retrieval and synthesis” process in the guideline development [27–29], thus aimed to collect and synthesize the evidence of the previously identified conditions and consult with experts. The optimization of the list includes two sub-steps: (1) evidence evaluation of each potential disease in the list by systematic review; and (2) a Delphi consensus survey to identify appropriate diseases to be added into China’s list of ACSCs.

#### Evidence evaluation

**Fig. 2:**
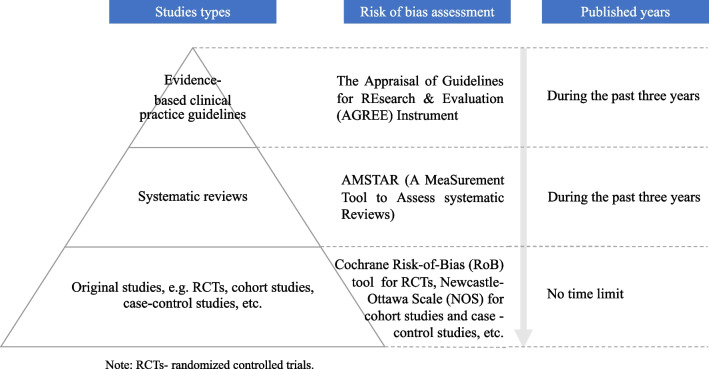
The evidence appraised principles

For each potential disease, we will conduct a systematic evidence search, selection, evaluation and grading according to the evidence appraised principles of Fig. 2, which is adapted from the 6S hierarchy of pre-appraised evidence [39]. The electronic databases are six commonly used Chinese and English databases, including CNKI, CBMdisc, Wanfang Database, MEDLINE (via PubMed), Scopus and Web of Science, and two evidence-based medical databases e.g., the Cochrane Library and Epistemonikos. There will be no publication status restrictions. The evidence evaluation and grading tools are the most commonly used ones, which match the corresponding study types, for example, the AMSTAR tool for systematic reviews [40]. To minimize potential bias, literature searching, screening, and evidence evaluation and grading will be performed by two reviewers in pairs independently. In addition, corresponding evidence summary forms will be created for each potential disease to be further investigated via Delphi consensus surveys.

#### Delphi consensus survey

The purpose of the Delphi consensus survey is to revise the ACSCs list based on expert experiences to further obtain face-to-face consensus. We will identify the respondents through online queries combined with recommendations from the Advisory Group, and build a multidisciplinary team of experts from all over the country, with an emphasis on including a higher proportion of clinicians as previously mentioned. We will conduct two rounds of surveys based on "Wenjuanxing" (https://www.wjx.cn/app/survey.aspx) to collect opinions and provide feedbacks to respondents. To eliminate possible interference, feedbacks will be anonymized with basic information of the respondents. Consistency in participants across both surveys is maintained, with those in the second round having the option to abstain due to special circumstances. Moreover, we will thoroughly evaluate potential conflicts of interest among participants to ensure that all respondents have no significant conflicts of interest.

The Delphi surveys focus on whether the diseases determined from preceding steps are really belong to the China’s ACSCs list, comprehensively considering existing evidence and real practice, and whether there are any other conditions that meet the criteria but have not yet been included. Detailed background information on this project will be explained in the questionnaire to prevent participants from distorting their opinions and answers. For each potential disease added into the ACSCs list, the respondents can exclusively select "agree", "disagree" or "not sure", or they can add other relevant important diseases and with further explanations on the underlying reasons. After the survey, the Coordination Team will summarize the survey results, collect all suggestions and comments, and revise and improve the ACSCs list according to those suggestions and comments. Diseases with more than 75% "agree" from respondents will be included in the list, and those with more than 50% "disagree" from respondents will be deleted. The ACSCs list will be listed in descending order according to the survey results.

### Step IV: approval of the final list version

We will organize a face-to-face consensus meeting to determine the final version of ACSCs list that contains diseases and codes applicable under the context of China’s healthcare system. Based on the results of the Delphi survey, the Coordination Team will compile the names, codes, evidence status, and the percentage of "agree" received from respondents for each disease included in the ACSCs list. We will create a final report to be disseminated among attendants for the consensus meeting. The Coordination Team will also determine the meeting time, duration (3–5 h), and location of the meeting, create the agenda and make relevant arrangements.

Prior to the meeting, the Advisory Group will conduct an updated assessment of potential conflicts of interest among all participants to ensure that no respondent has a significant conflict of interest. At the meeting, the chairman will introduce the participants and the meeting topic, provide the documents to be published with related information and evidence. Meanwhile, a brief introduction will be given to describe the main purposes and processes of the development of the ACSCs list in details. All participants, especially members of the Coordination Team and the Advisory Group, will need to review and reach consensus (≥ 75%) on the contents of the final version of the list through consensus discussion, which will be further disseminated via peer-reviewed journals, newspapers, academic conferences and other approaches.

#### Patient and public involvement

Patient preferences and values are a major factor when it comes to selecting health services. The evaluation of medical quality not only considers the accessibility of health services and health outcomes, but also includes the patient's experience and satisfaction. To account for the preferences and values of patients and the public in seeking health services, we will include two or three relevant representatives in the face-to-face consensus process. Our strategy for participant enrollment entails the use of convenient sampling, targeting individuals with common or widespread diseases and their caregivers. However, due to logistical limitations, it is not possible to enlist representative patients for each specific condition. Consequently, we will favor those with enhanced health literacy to ensure the ease of gathering their insights.

## Discussion

This project aims to develop the first version of ACSCs tailored for China’s healthcare system, drawing on international experiences and evidence-based medicine. To achieve this, we will adopt a systematic and rigorous methodology that incorporates multidisciplinary expertise, including clinical medicine, epidemiology, health policy making, evidence-based medicine, economics, and more.

### Our study in the context of previous research

The hospitalization rate of ACSCs has been proposed by previous literature as a negative indicator to evaluate the accessibility and quality of health services [2]. Numerous studies in this field have attempted to refine the definition of ACSCs and its evaluation criteria, recognizing that timely provision of effective ambulatory care can significantly alleviate and control symptoms of certain medical conditions as an early-stage intervention, potentially reducing or avoiding subsequent inpatient services.

Determining which diseases belong to ACSCs usually begins with a literature review, which attempts to identify diseases potentially associated with ambulatory service delivery. Clinical professionals, especially primary care physicians, would review the list created in the previous step according to multiple methodologies and standards [41, 42]. The main inclusion considerations for ACSCs are described as follows: (1) determine if similar indicators have been used in previous studies; (2) make sure the conditions under consideration are significant health concerns and clinically relevant to ambulatory care issues; (3) check if the conditions are coded in a large population-based data source; (4) emphasize the importance of preventing disease onset and admissions, as well as ensuring timely admission within 48 h when needed [35, 41].

To date, many countries and institutions have developed their own ACSCs lists [8-10] under different contexts, which typically include diabetes, hypertension, congestive heart failure, chronic obstructive pulmonary disease, asthma, etc. Additionally, a growing body of research has been conducted concerning the application and evaluation of ACSCs, not only for research purposes, but also for the practical application among national health systems and international organizations as an essential indicator of performance.

### Implications for practice and research

China’s list of ACSCs is expected to serve as an essential indicator of healthcare quality and accessibility and will provide evidence-informed recommendations for decision-making. On the one hand, hospitalization rates of ACSCs can be compared across regions and population groups to identify access and capacity disparities. This information can identify major areas for improvement in order to reduce potentially avoidable hospitalizations. On the other hand, it can serve as a statistical indicator for monitoring trends and evaluating service continuity. Additionally, the ACSCs lists can be used to determine whether disasters will have a detrimental effect on public health and healthcare systems, as well as to identify vulnerable groups [43, 44]. Furthermore, ACSCs can measure changes in hospitalization rates before and after policy interventions to evaluate their effectiveness.

The development of ACSCs in China is a crucial step towards value-based healthcare. The experiences might be broadly applicable, particularly to the leading BRICS emerging nations, which are driving real GPD growth and global demand for medical goods and services [45, 46]. China’s success in this area could also serve as an example for neighboring health systems in Asia, both within and outside of the OECD [47].

### Strengths and limitations

Our proposal has several strengths. Currently, the existing international lists of ACSCs are primarily developed through expert consultation using the Delphi method or modified Delphi method. This project innovatively extends the development method of clinical practice guidelines to the development of ACSCs lists. To minimize potential bias in the development process, we will implement certain quality control measures at each step. For example, during the scoping review and systematic review process, two independent reviewers will verify the accuracy of the results. In the Delphi consensus survey and face-to-face consensus, we will gather opinions from various stakeholders. We will also ensure diversity among participants in terms of geographical locations, professional titles and gender to enhance the comprehensiveness and representativeness of suggestions. Moreover, the approach proposed in this study optimally balances rigorous stakeholder engagement and a comprehensive review of literature and local evidence, aiming to maximize the likelihood of its adoption by the policy and practice communities.

Our proposal also has some limitations. At this stage, our study is limited to identifying diseases for inclusion in China’s ACSCs list, and we will not be conducting an extensive examination of the accuracy, reliability, and validity of the list’s assessment of medical performance in practice. Future studies are expected to conduct in-depth investigations in these aspects to facilitate the optimization of the list.

## Data Availability

Not applicable because a protocol should not contain any data; it sets out the research questions and how they will be addressed.

## Abbreviations

ACSCs: Ambulatory care sensitive conditions
AHRQ: Agency for healthcare research and quality
CBMdisc: China biology medicine disc
CIHI: Canadian institute for health information
CNKI: China national knowledge internet
IOM: Institute of medicine
NICE: National institute for health and care excellence
OECD: Organization for economic co-operation and development
WHO: World Health Organization
